# Novel associations of *GSN* indel and *PLAG1* c.48333G > A variants with growth traits in mature Indonesian Madura and Bali cattle

**DOI:** 10.5455/javar.2026.m1026

**Published:** 2026-03-16

**Authors:** Hartati Hartati, Widya Pintaka Bayu Putra, Mariyono Mariyono, Muhammad Luthfi, Lukman Affandhy, Dewi Khosiya Robba, Eko Handiwirawan, Sulistiyoningtiyas Irmawanti, Aryogi Aryogi, Susan Maphilindawati Noor, Endang Romjali, Wike Andre Septian

**Affiliations:** 1 Research Center for Animal Husbandry, National Research and Innovation Agency (BRIN), Cibinong, Bogor, Indonesia; 2 Research Center for Applied Zoology, National Research and Innovation Agency (BRIN), Cibinong, Bogor, Indonesia; 3 Hydrodynamics Laboratory, National Research and Innovation Agency (BRIN), Cibinong, Bogor, Indonesia; 4 Research Center for Veterinary Science, National Research and Innovation Agency (BRIN), Cibinong, Bogor, Indonesia; 5 Animal Biotechnology, Faculty of Animal Science, Brawijaya University, Malang, Indonesia

**Keywords:** Bali cattle, gene polymorphism, growth traits, *GSN*, Madura cattle, *PLAG1*

## Abstract

**Objectives:** This study aimed to identify a 21 bp insertion/deletion mutation in the *GSN* gene (intron 2) and a single nucleotide polymorphism (c.48333G > A) in the *PLAG1* gene (exon 3), and evaluate their association with growth traits in mature Madura and Bali cattle.

**Materials and Methods:** A total of 219 animals (156 Madura and 73 Bali cattle) were assessed for body weight (BW) and four body measurements: Heart girth (HG), body length (BL), hip height (HH), and withers height (WH). Genotyping was performed using PCR and PCR-RFLP techniques.

**Results:** Both genes were polymorphic, with high PIC values (> 0.30). The *GSN* gene polymorphism was significantly associated with HH in Bali cattle, while the *PLAG1* gene showed significant associations with BW in both breeds.

**Conclusions:** These findings suggest that *PLAG1* (c.48333G > A) is a promising genetic marker for BW in Indonesian tropical cattle.

## 1. Introduction

Madura (*Bos indicus*) and Bali (*Bos javanicus*) cattle are two indigenous breeds in Indonesia with great potential to contribute to the national beef industry due to their adaptability and genetic uniqueness [1, 2]. Madura cattle are believed to result from historical hybridization between *B. indicus* and *B. javanicus*, as confirmed by single-nucleotide polymorphism (SNP) array analysis [3]. Bali cattle, on the other hand, are known for their resilience to tropical environments and their suitability for low-input farming systems [4]. However, the productivity of these breeds still faces several challenges, including relatively slow growth rates and suboptimal feed efficiency. Additionally, limitations in genetic breeding have resulted in considerable variation in growth and body size among individuals within the population. Therefore, efforts to enhance the productivity of Madura and Bali cattle through genetically-based approaches are becoming increasingly important to support food security and the livestock industry in Indonesia.

In recent years, the integration of molecular genetics into animal breeding has opened new avenues for improving livestock performance. Marker-assisted selection (MAS), which utilizes genetic polymorphisms linked to desirable traits, enables early and accurate identification of superior animals, thereby accelerating the pace of genetic progress [5, 6, 7]. Among candidate genes associated with growth traits, Gelsolin (*GSN*) and Pleomorphic Adenoma Gene 1 (*PLAG1*) have attracted attention for their roles in cellular growth regulation, cytoskeletal remodelling, and metabolic activity [8, 9].

The *GSN* gene is located on chromosome 8, spanning 60,994 bp with 23 exons, while the *PLAG1* gene is located on chromosome 14, spanning 51,970 bp with four exons (GenBank: NC_037335.1; NC_037341.1). Previous studies have reported that mutations in the *GSN* gene, such as a 21 bp indel in intron 2, affect cattle growth in several Chinese cattle populations [7]. While the bovine *PLAG1* gene had many mutation sites with a significant effect for growth traits of cattle, such as c.48333G > A [10, 11], g.25015640G > T [12, 13], c.957A > C [14], and an indel 19 bp mutation [15]. However, research on the role of *GSN* and *PLAG1* gene polymorphisms in the growth performance of Madura and Bali cattle remains limited, necessitating further investigation to uncover the genetic potential of these genes in molecular selection programs.

Therefore, this study aimed to identify polymorphisms in the *GSN* and *PLAG1* genes in Madura and Bali cattle and to analyze their association with growth traits, specifically body weight and body measurements, at maturity. We hypothesize that polymorphisms in the *PLAG1* gene are significantly associated with body weight, while *GSN* polymorphisms may influence body size traits. Findings from this study are expected to contribute to the development of effective molecular selection strategies for Indonesia’s tropical cattle.

## 2. Materials and Methods

### 2.1. Ethical approval

This study was approved by the Ethics Committee for the Maintenance and Use of Experimental Animals, National Research and Innovation Agency (Approval Number: 153/KE.02/SK/07/2023).

### 2.2. Experimental animals and DNA extraction

A total of 219 mature cattle (> 3 years old), consisting of 156 Madura and 73 Bali cattle of both sexes, were used. 5 mm of whole blood was collected from the jugular vein using EDTA-coated vacutainer tubes. DNA was extracted using a commercial genomic DNA extraction kit (Geneaid, Taiwan) following the manufacturer’s instructions.

### 2.3. Research site

The animals were obtained from the Indonesian Cattle Research Institute located in Grati District, Pasuruan Regency, East Java Province, Indonesia. The geographical coordinates of Pasuruan Regency are 112°33’55” – 113°05’37” E and 7°32’34” – 7°57’20” S, with elevations ranging from 25 to 100 m above sea level. The region has an average temperature of 21.92°C, relative humidity of 81.67%, and annual rainfall of 3,072.3 mm.

### 2.4. Animal management

The cattle were housed in group pens under a natural mating system, with each bull managing 15–20 cows. The basal diet consisted primarily of Elephant grass (*Pennisetum purpureum*) (97.98%) and rice straw (2.02%). The concentrate diet was formulated with the following composition: Chalk (1.89%), salt (1.89%), rice bran (24.75%), cracked corn (20.51%), coffee husk (4.98%), palm kernel cake (9.70%), copra meal (10.16%), cassava flour (10.16%), dried distillers grains with solubles (7.98%), and corn gluten feed (7.98%). The formulated diet contained approximately 9–11% crude protein (CP), 58–60% total digestible nutrients (TDN), and 17–22% crude fiber (CF).

### 2.5. Phenotypic measurements

Body weight (BW) was measured using an electronic digital scale. Four linear body measurements, including heart girth (HG), body length (BL), hip height (HH), and withers height (WH), were obtained for each animal following the protocol described by Tyasi and Putra [16].

### 2.6. PCR amplification

The target regions of *GSN* and *PLAG1* genes were amplified in a 15 μl PCR reaction mixture containing 1.5 μl of genomic DNA, 0.3 μl of each primer, 7.5 μl of PCR master mix, and 5.4 μl of nuclease-free water. The PCR cycling conditions included initial denaturation at 95°C for 5 min, followed by 35 cycles of denaturation at 95°C for 15 sec, annealing at 57°C for 30 sec, extension at 72°C for 45 sec, and a final extension at 72°C for 5 min. PCR products were visualized by 2% agarose gel electrophoresis at 100 V for 30 min, then viewed under UV transillumination. The primer pairs used in this study are presented in Table 1.

**Table 1.** Primer sequences used for PCR amplification of the *GSN* and *PLAG1* genes in Madura and Bali cattle.

Gene	Primer	Size (bp)	Region	GenBank	Reference
*GSN*	F: 5′-AGA TGA GGC GGG TGA TTA GG -3′	270/249	Intron 2	NC_037335.1	[7]
	R: 5′- GTC CTT TCC CCC TCT CAA ACA -3′				
*PLAG1*	F: 5′- CCT TTG CCT GTT GCT TTC CC -3′	628	Exon 3	NC_037341.1	[10]
	R: 5′- GCG CGT ATC AGT CAG GAC AT -3′				

### 2.7. Genotyping

Detection of mutation site in the *GSN* and *PLAG1* genes was performed in four Madura cows (1 head/genotype/gene) using a sequencing analysis by Apical Scientific (Malaysia). According to the *GSN* gene amplification, animals can be identified as having the DD (249 bp), II (270 bp), or ID (249 bp; 270 bp) genotypes. While the PCR-RFLP analysis of the *PLAG1/SacII* gene was performed in animals under study, with the reaction composition per sample of 5 μl of PCR product, 0.4 μl of SacII restriction enzyme (CCGC*GG), 0.7 μl of buffer 10x, and 0.9 μl of nuclease-free water. According to the *PLAG1/SacII* gene polymorphism, cattle can be identified as AA (628 bp), GG (106 bp; 522 bp), and AG (104 bp; 524 bp; 628 bp) genotypes.

### 2.8. Data analysis

The effect of sex was reduced using a mathematical formula referring to Hardjosubroto [17] as follows:


\[
{\mathrm{C}}{{\mathrm{F}}_{{\mathrm{sex}}}}{ = }{\mathrm{A}}{{\mathrm{P}}_{\mathrm{M}}}/{\mathrm{A}}{{\mathrm{P}}_{\mathrm{F}}}
\]

\[
{\mathrm{C}}{{\mathrm{P}}_{\mathrm{F}}}{ = }{\mathrm{\;A}}{{\mathrm{P}}_{\mathrm{F}}}{ \times }{\mathrm{C}}{{\mathrm{F}}_{{\mathrm{sex}}}}
\]

Where, CF_sex_ is the correction factor of sex; CP_F_ is the corrected performance of female; AP_M_ is the actual performance of male and AP_F_ is the actual performance of female. Therefore, the genetic diversity param such as: Genotype frequency, allele frequency, observed heterozygosity (H_o_), expected heterozygosity (H_e_), number of effective allele (n_e_), polymorphic informative content (PIC) and *Chi*-square (χ^2^)) values were calculated according to Nei and Kumar [18]. Therefore, the association study between gene polymorphism with body weight and measurement was computed using General Linear Model (GLM) with a mathematical formula as follows:


\[
{{\mathrm{Y}}_{\mathrm{i}}}\;\;{ = \mu + }{{\mathrm{G}}_{\mathrm{i}}}{ + }{{\mathrm{E}}_{{\mathrm{ij}}}}
\]

where, Y_i_ is the observation value; μ is the common means; G_i_ is the effect of i^th^ genotype; and E_ij_ is the experimental error.

## 3. Results

### 3.1. Genotyping of GSN and PLAG1 genes

**Figure 1.**
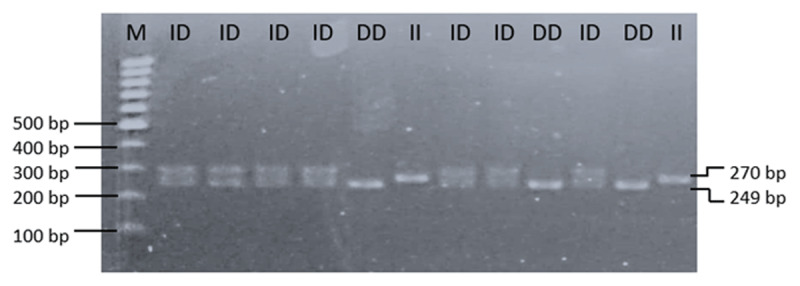
Genotyping of the bovine *GSN* gene (intron 2) with PCR analysis reveals three genotypes of DD (249 bp), II (270 bp), and ID (249 bp and 270 bp). M: DNA ladder 100 bp.

**Figure 2.**
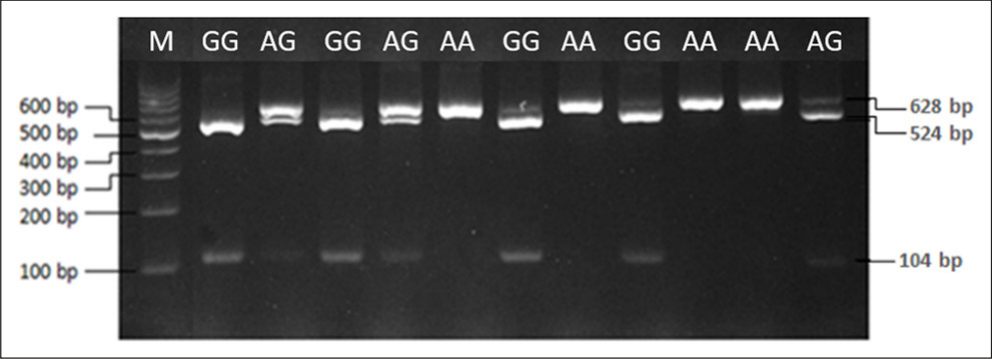
Genotyping of the bovine *PLAG1/SacII* gene (exon 3) with PCR-RFLP analysis reveals three genotypes of GG (104 bp and 524 bp), AA (628 bp), and AG (104 bp, 524 bp, and 628 bp). M: DNA ladder 100 bp.

**Figure 3.**
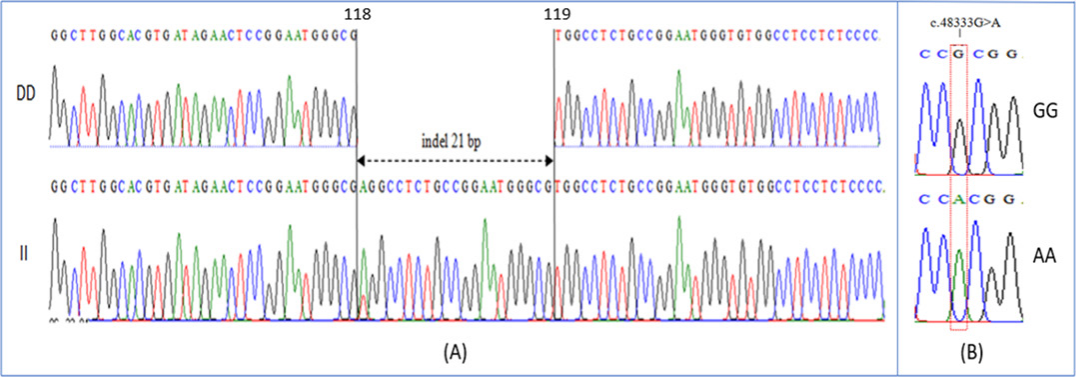
Detection of mutation sites in the *GSN* and *PLAG1* genes of cattle. A: a mutation of g.118_119indel.21 in the intron 2 region of bovine *GSN* gene (GenBank: PP339753.1/PP339754.1). B: a mutation of c.48333G > A in the exon 3 region of *bovine PLAG1* (GenBank: NC_037341.1).

Along 249/270 bp of *GSN* gene was successfully amplified on 2% agarose gel (Figure 1). Hence, three genotypes of DD, II and ID were observed in the Madura and Bali cattle under study. Therefore, the results of PCR-RFLP in *PLAG1/SacII* gene reveal three genotypes of AA, GG and AG (Figure 2). In this study, the sequencing results in *GSN* gene of Madura cattle were deposited in the NCBI database with accession of GenBank: PP339753.1 for deletion type and PP339754.1 for insertion type. While, the sequencing results in *PLAG1* gene of Madura cattle under study were also deposited with accession of GenBank: PP339755 for GG genotype and PP339756.1 for AA genotype. According to the deposited sequence, the indel 21 bp mutation of bovine *GSN* gene was occurred between 118^th^ and 119^th^ nucleotide (g.118_119indel.21). Subsequently, a transition mutation (c.48333G > A) was confirmed in the restriction site of *SacII* in bovine *PLAG1* gene (Figure 3).

### 3.2. Genetic diversity

In Madura cattle, ID (0.51) and AA (0.46) are the common genotypes in *GSN* and *PLAG1* genes (Table 2). While, II and ID were detected as the common genotype (0.40) in *GSN* gene of Bali cattle. Otherwise, the AA genotype (0.48) of *PLAG1/SacII* gene was more frequent in Bali cattle (Table 3). Commonly, the frequency of I allele was higher than D allele in the *GSN* gene polymorphism for both breeds. In addition, the *PLAG1/SacII* gene polymorphism of both breeds had the higher of A allele rather than G allele. Subsequently, the PIC value in *GSN* and *PLAG1* genes for both breeds were classified as high category (> 0.30). The polymorphism of *GSN* gene in both breeds was under the genetic equilibrium. However, the polymorphism of *PLAG1* gene in Bali cattle was under the genetic equilibrium and it was not observed in Madura cattle.

**Table 2.** Genotype and allele frequencies, heterozygosity indices, and PIC values for *GSN* and *PLAG1* gene polymorphisms in Madura cattle (*Bos indicus*).

Parameter	*GSN*	*PLAG1*
Genotype frequency (N)	DD = 0.17 (27)	AA = 0.46 (63)
	II = 0.32 (50)	GG = 0.41 (57)
	ID = 0.51 (79)	AG = 0.13 (18)
Allele frequency	I = 0.57	A = 0.52
	D = 0.43	G = 0.48
Observed heterozygosity (H_o_)	0.51	0.13
Expected heterozygosity (H_e_)	0.49	0.50
Polymorphic informative content (PIC)	0.37	0.37
Number of effective alleles (n_e_)	1.96	2.00
Chi-square (χ^2^)	0.19*	75.29

N: number of observations; *under the genetic equilibrium

**Table 3.** Genotype and allele frequencies, heterozygosity indices, and PIC values for *GSN* and *PLAG1* gene polymorphisms in Bali cattle (*Bos javanicus*).

Parameters	*GSN*	*PLAG1*
Genotype frequency (N)	DD = 0.20 (15)	AA = 0.48 (20)
	II = 0.40 (29)	GG = 0.32 (8)
	ID = 0.40 (29)	AG = 0.20 (13)
Allele frequency	I = 0.60	A = 0.65
	D = 0.40	G = 0.35
Observed heterozygosity (H_o_)	0.40	0.32
Expected heterozygosity (H_e_)	0.48	0.46
Polymorphic informative content (PIC)	0.37	0.35
Number of effective alleles (n_e_)	1.93	1.84
Chi-square (χ^2^)	2.24*	3.85*

N: number of observations; *under the genetic equilibrium

### 3.3. Association analysis

The polymorphism of the *PLAG1* gene showed a significant association with the body weight (BW) of Madura (Table 4) and Bali (Table 5) cattle at maturity age (*p* < 0.05). While, the polymorphism of *GSN* gene had the significance association with hip height (HH) of Bali cattle at maturity age (Table 5). In Madura cattle, the GG genotype (*PLAG1*) had lower body weight than the AA/GG genotype (*p* < 0.05). In contrast, the GG genotype (*PLAG1*) in Bali cattle showed higher body weight than the AG genotype. In addition, the polymorphism of *GSN* gene in the present study showed a significant association with hip height (HH) of Bali cattle. In this case, the ID genotype had lower HH than the DD genotype. Nonetheless, the *GSN* gene polymorphism was not significantly associated with BW in Madura and Bali cattle. Moreover, the body measurements of Madura cattle were not influenced by *GSN* gene polymorphism.

**Table 4.** Least square means ± SD of body weight and linear body measurements across different genotypes of *GSN* and *PLAG1* genes in Madura cattle (> 3 years old).

Gene	Genotype	Performance				
		BW	HG	BL	HH	WH
*GSN*	DD	271.69 ± 91.35 (18)	148.92 ± 20.31 (24)	117.52 ± 16.52 (24)	119.00 ± 8.19 (17)	114.52 ± 10.36 (24)
	II	271.00 ± 50.92 (27)	152.92 ± 12.71 (40)	120.75 ± 10.25 (40)	118.33 ± 4.90 (26)	117.55 ± 6.49 (40)
	ID	276.27 ± 70.52 (58)	152.28 ± 16.22 (74)	119.32 ± 12.48 (74)	120.17 ± 7.91 (58)	116.30 ± 15.24 (74)
*PLAG1*	AA	281.40 ± 78.39^a^ (43)	151.21 ± 17.18 (57)	119.92 ± 13.10 (57)	120.80 ± 7.92 (43)	117.75 ± 9.02 (57)
	GG	233.05 ± 41.22^b^ (10)	144.12 ± 15.10 (17)	117.76 ± 12.69 (17)	120.84 ± 7.05 (10)	116.68 ± 11.15 (17)
	AG	278.55 ± 58.06^a^ (31)	151.88 ± 14.79 (44)	120.89 ± 12.80 (44)	120.03 ± 5.91 (27)	117.26 ± 7.51 (44)

N: number of animals; BW: body weight (kg); HG: heart girth (cm); BL: body length (cm); HH: hip height (cm); WH: withers height (cm); ^a,b^, superscript in the different columns differ significantly (*p* < 0.05)

**Table 5.** Least square means ± SD of body weight and linear body measurements across different genotypes of *GSN* and *PLAG1* genes in Bali cattle (> 3 years old).

Gene	Genotype	Performance				
		BW	HG	BL	HH	WH
*GSN*	DD	320.80 ± 105.70 (15)	170.00 ± 23.11 (15)	122.63 ± 15.21 (15)	119.87 ± 8.39^a^ (15)	118.73 ± 8.84 (15)
	II	300.44 ± 56.22 (27)	163.09 ± 16.63 (27)	117.59 ± 9.80 (27)	115.13 ± 7.16^ab^ (27)	115.04 ± 6.19 (27)
	ID	303.39 ± 98.59 (27)	165.48 ± 20.14 (27)	117.11 ± 13.95 (27)	114.40 ± 9.10^b^ (27)	115.10 ± 10.22 (27)
*PLAG1*	AA	285.24 ± 47.31^ab^ (17)	158.88 ± 19.20 (17)	115.50 ± 8.47 (17)	114.20 ± 6.81 (17)	113.80 ± 6.06 (17)
	GG	328.64 ± 71.53^a^ (7)	169.43 ± 8.94 (7)	118.29 ± 7.76 (7)	113.14 ± 1.52 (7)	114.36 ± 5.65 (7)
	AG	274.00 ± 45.92^b^ (13)	161.88 ± 8.91 (13)	117.27 ± 11.11 (13)	114.15 ± 7.17 (13)	115.14 ± 6.72 (13)

N: number of animals; BW: body weight (kg); HG: heart girth (cm); BL: body length (cm); HH: hip height (cm); WH: withers height (cm); ^a,b^, superscript in the different columns differ significantly (*p* < 0.05)

## 4. Discussion

The PIC value had three categories: low (< 0.10), moderate (0.10–0.30), and high (> 0.30), as stated by Nei and Kumar [18]. The PIC value for the *GSN* and *PLAG1* genes of the experimental animals was classified as a high category. Hence, both genes have the potential to be candidate genes for growth traits in Madura and Bali cattle. Zhong et al. [10] reported that the homozygous AA Chinese cattle had the lowest body measurements, and the homozygous GG genotype had the highest body measurements. In this study, the polymorphism of the *PLAG1* gene in Madura cattle was not in genetic equilibrium, which could have been caused by the selection program at the breeding station. Despite this, migration, inbreeding, crossbreeding, and geographical situation may affect the genetic equilibrium of animals [19].

The *GSN* and *PLAG1* genes in Madura and Bali cattle were shown to be polymorphic and have the potential to improve growth traits through molecular selection. In the chickens (*Gallus domesticus*), the *GSN* gene influences embryo development [20]. Napolitano et al. [21] obtained nine mutation sites in the *ovine GSN* gene. Interestingly, two mutation sites in intron 12 (g.2003C > T and g.2103G > A) showed a significant association with milk quality traits in Italian sheep. In humans (*Homo sapiens*), two missense mutations in the *GSN* gene (p.D187N and p.Y447H) can affect the cranial and peripheral neuropathy, carpal tunnel syndrome, and autonomic dysfunction [22]. Previously, a 21 bp indel mutation in the *GSN* gene was found to be significantly associated with body measurements in Chinese cattle breeds [7]. In that study, the homozygous II Jiaxian cattle had the highest WH, BL, and HG measurements, similar to those of Madura cattle in the present study.

Specifically, the *PLAG1/SacII* gene was significantly associated with BW in Madura and Bali cattle. Putra et al. [14] reported that the *PLAG1/TaqI* (c.795A > G) gene in exon 2 was not significantly associated with the body weight of Bali cattle. In goats (*Capra hircus*), an indel deletion of 15 bp in the *PLAG1* gene has been detected and is significantly associated with growth traits [23]. Otherwise, two indel mutations of 30 bp and 45 bp were found in the sheep *PLAG1* gene and were significantly associated with body measurements [24].

The significant associations observed in this study suggest that these polymorphisms could serve as potential genetic markers for improving growth traits in Madura and Bali cattle through molecular selection. However, genetic traits do not act in isolation; environmental factors such as feed quality [25], management practices [26], and climate conditions [27] also influence growth performance. Future studies should incorporate gene-environment interactions to determine the extent to which genetic variations interact with external factors in shaping cattle phenotypes.

From a practical perspective, integrating molecular selection based on *PLAG1* and *GSN* polymorphisms into breeding programs could enhance the genetic improvement of Madura and Bali cattle. Given that the Indonesian government has implemented breeding initiatives through Village Breeding Centers, these findings could be applied to refine selection criteria for superior growth traits. The use of genomic information in breeding programs can facilitate the development of tropically adapted composite breeds, thereby enhancing genetic improvement and the conservation of local cattle breeds [28].

## 5. Conclusions

This study confirms that the *PLAG1* (c.48333G > A) and *GSN* (21 bp indel) gene polymorphisms play a significant role in the growth traits of Madura and Bali cattle. The *PLAG1* gene was strongly associated with body weight, while the *GSN* gene influenced hip height in Bali cattle. These findings provide valuable insights for the implementation of molecular selection programs to enhance the genetic potential of tropical cattle. Despite the promising results, further validation with larger sample sizes and functional genomic studies is necessary to fully understand the impact of these polymorphisms on growth traits. The integration of genetic selection into breeding programs can contribute to more efficient and sustainable cattle production in Indonesia, supporting national efforts to improve livestock productivity and food security.

## Data Availability

The data presented in this study are available from the corresponding author upon reasonable request.

## List of abbreviations

AA, Homozygous genotype A/A; AG, Heterozygous genotype A/G; BL, body length; bp, base pair; BRIN, National Research and Innovation Agency (Indonesia); BW, body weight; CF, crude fiber; cm, centimeter; CP, crude protein; °C, degree Celsius; DD, Homozygous deletion genotype; DNA, Deoxyribonucleic acid; EDTA, Ethylenediaminetetraacetic acid; gm, gram; GG, Homozygous genotype G/G; *GSN*, Gelsolin gene; HG, Heart girth; HH, Hip height; Ho, Observed heterozygosity; He, Expected heterozygosity; ID, Insertion–deletion heterozygous genotype; II, Homozygous insertion genotype; indel, Insertion/deletion mutation; kg, kilogram; m, meter; MAS, Marker-assisted selection; min, minutes; mm, millimeter; μl, microliter; NCBI, National Center for Biotechnology Information; ne, Number of effective alleles; PCR, Polymerase chain reaction; PCR-RFLP, Polymerase chain reaction–restriction fragment length polymorphism; PIC, Polymorphic information content; *PLAG1*, Pleomorphic adenoma gene 1; rpm, revolutions per minute; SNP, Single nucleotide polymorphism; TDN, total digestible nutrients; UV, Ultraviolet; V, Volt; WH, Withers height; χ^2^, *Chi*-square value.
